# Mie scatter spectra-based device for instant, contact-free, and specific diagnosis of bacterial skin infection

**DOI:** 10.1038/s41598-017-05061-1

**Published:** 2017-07-06

**Authors:** Robin E. Sweeney, Elizabeth Budiman, Jeong-Yeol Yoon

**Affiliations:** Biomedical Engineering Graduate Interdisciplinary Program and Department of Biomedical Engineering, The University of Arizona, Tucson, Arizona 85721 United States

## Abstract

Rapid and specific diagnostic techniques are needed to expedite specific treatment of bacterial skin infections with narrow-spectrum antibiotics, rather than broad-spectrum. Through this work a device was developed to determine the presence of and species responsible for a bacterial skin infection using differences in Mie scatter spectra created by different bacterial species. A 650 nm LED at five different incident angles is used to illuminate the tissue, with Mie scatter being detected by PIN photodiodes at eight different detection angles. Mie scatter patterns are collected at all photodiode angles for each of the incident light angles, resulting in a Mie scatter spectra. Detectable differences in Mie scatter spectra were found using the device developed between commensal bacteria (no infection) and bacteria inoculated (infection) on the surface of both porcine and human cadaveric epidermis. Detectable differences were found between species of infection, specifically *Escherichia coli* and *Staphylococcus aureus*, with differences summarized through principle component analysis. Mie scatter spectra can be detected within a few seconds without skin contact. This device is the first to rapidly and specifically diagnose bacterial skin infections in a contact-less manner, allowing for initial treatment with narrow spectrum antibiotics, and helping to reduce the likelihood of resistance.

## Introduction

Skin, both human and animal, is covered with bacteria continually^1–5^. Typically these bacteria are commensal, or healthy bacteria. When commensal bacteria become out of balance, or pathogenic species and strains of bacteria are added, an infection results^3, 5–7^. A skin infection typically results in a rash for healthy human and animal subjects, but can be limb or life threatening for some subjects^6–9^. Bacterial skin infections may cause disease states such as impetigo, cellulitis, abscesses, necrotizing fasciitis, and other severe health issues^8–11^. As the skin acts as a protective barrier to the body, any minor break in the skin combined with a skin infection can lead to deep tissue infections, bone infections, sepsis, and even death^6, 7^.

Bacterial skin infections are a major concern with risk of community acquired infections as well as nosocomial infections^7, 12^. Risk factors that increase the likelihood of bacterial skin infections include diabetes, poor immune function (including immunocompromised patients or children with immature immune systems), use of immunosuppressive drugs, immobility, extended hospital stays, poor hygiene, and poor circulation to extremities^4, 6, 8, 9, 11, 13^.

Common bacterial species that cause skin infections include *Staphylococcus aureus*, *Streptococcus pyogenes, Pseudomonas aeruginosa*, and *Escherichia coli* ^6–11^. A large diagnostic hurdle is the fact that many of these bacteria, particularly *S. aureus*, are commonly found on the skin as commensal bacteria^2–5, 14–16^. A major issue with common skin infections is the prevalence of antibiotic resistance in common pathogens, namely, *S. aureus*. Antibiotic resistance is a major health issue, with MRSA and VRSA (methicillin resistant *S. aureus* and vancomycin resistant *S. aureus*, respectively) being some of the biggest issues currently^7, 10, 15, 16^.

Only single-species bacterial infection was demonstrated in this work, in the presence of commensal bacteria, since a large majority of bacterial skin infection cases, are single species infections. In a large study where data was compiled for 471,550 skin and soft tissue infections (SSTI), only 6% of the cases where a pathogen was isolated showed multiple pathogens in a single SSTI^17^. Multi-species infection primarily occurs in chronic infections, untreated cases of skin infection, or in instances where antibiotic treatment has been previously administered^18^, which is a rare case and not a common bacterial skin infection situation. Nonetheless, our device aims to prevent this situation from becoming a reality for patients through early and frequent monitoring.

The best approach to treating a bacterial infection is through multiple, narrow-spectrum antibiotics specific to the species and strain of bacteria responsible for the infection, which is only possible once a pathogen has been identified^6, 8, 19^. Diagnostic methods using bacterial culture techniques can take significant amounts of time to determine a specific bacterial species and strain, resulting in a delay in the administration of optimal treatments. Reducing the time to specific diagnosis would reduce the time to specific and effective treatment, increasing the efficacy of these treatments and helping to slow the evolution of antibiotic resistant bacteria.

Currently the gold standard diagnostic measure for determining the specific bacterial species and strain responsible for a skin infection is bacterial culture using selective and differential media to determine the exact pathogen^7, 19^. Culture techniques take a minimum of many hours, but typically can take multiple days to specifically diagnose the nature and cause of an infection. In addition to the time required to diagnose patients via bacterial cultures, these techniques require trained staff, dedicated facilities, expensive equipment, and high reagent (media) costs. While waiting for the results of these diagnostic tests, patients are typically prescribed broad-spectrum antibiotics, which are known to heavily contribute to antibiotic resistance^15, 16^.

While diagnosis through bacterial culture is the current gold standard, other methods have been developed in recent years. Genomic diagnostic techniques are commonly being used, such as gene amplification via polymerase chain reaction (PCR), or gene sequencing of DNA or 16S ribosomal RNA^1, 2, 5, 20^. While these methods are effective at identifying specific bacterial species and even specific antibiotic susceptibilities or resistances, they are costly and time consuming. Mie scatter imaging systems, often called BARDOT (Bacterial Rapid Detection using Optical Scattering Technology) systems, have been used to identify bacterial species, but only following culture of the bacteria and not specifically for the purpose of diagnosing skin infection^21–23^. BARDOT systems not only require bacteria to be cultured before identification of the specific species is possible, but they require that bacterial colonies be on an optically transparent substrate, therefore making it impossible to translate this technology direction for use as a direct detection method from human skin^21–24^. All of these methods have similar issues in a clinical setting; they are time consuming, costly, and require specific (often expensive) laboratory equipment and a highly skilled staff.

A faster and more affordable approach to infection diagnosis is necessary to reduce health care costs, decrease time to treatment, and increase efficacy of treatment across a wide variety of populations. A mobile and rapid diagnostic device is essential to warn patients of infection early and even without repeated trips to a physician’s office or a hospital. Mobile monitoring would allow early notification of infection to a patient and a physician to allow early treatment and, therefore, improved efficacy of treatment, prognosis, and quality of life. Rapid optical analysis of skin infection eliminates the need for any reagents, lengthy assay times, tedious sample handling, and the need for access to skilled technicians in a laboratory.

In this work, we utilize Mie scatter spectra to directly detect infection from animal and human skin models. Mie scatter spectra are defined here as the collection of photodiode readings at eight different detection angles. Mie scatter patterns are collected across a variety of incident light angles (five different angles from the surface of each tissue sample). Mie scatter is dependent on particle size, morphology, refractive index, and concentration. Changes in these factors due to bacterial growth and interactions with tissue components, especially lipids, result in changes in scatter patterns across a variety of angles^25^. Our device takes advantage of changes in Mie scatter spectra based on these factors. In this work, we utilize Mie scatter spectra collected from various skin models, of both porcine and human skin, to rapidly detect bacterial infections on tissue surfaces and determine the bacterial species responsible for the infection. Unlike other Mie scatter-based biosensing techniques, such as the use of Mie scatter intensities at fixed angle to detect bacterial presence through particle immunoagglutination, this method does not require sample processing, additional reagents, or bioreceptor use, which eliminates time and labor for lengthy reagent and sample preparation^26, 27^.

**Figure 1:**
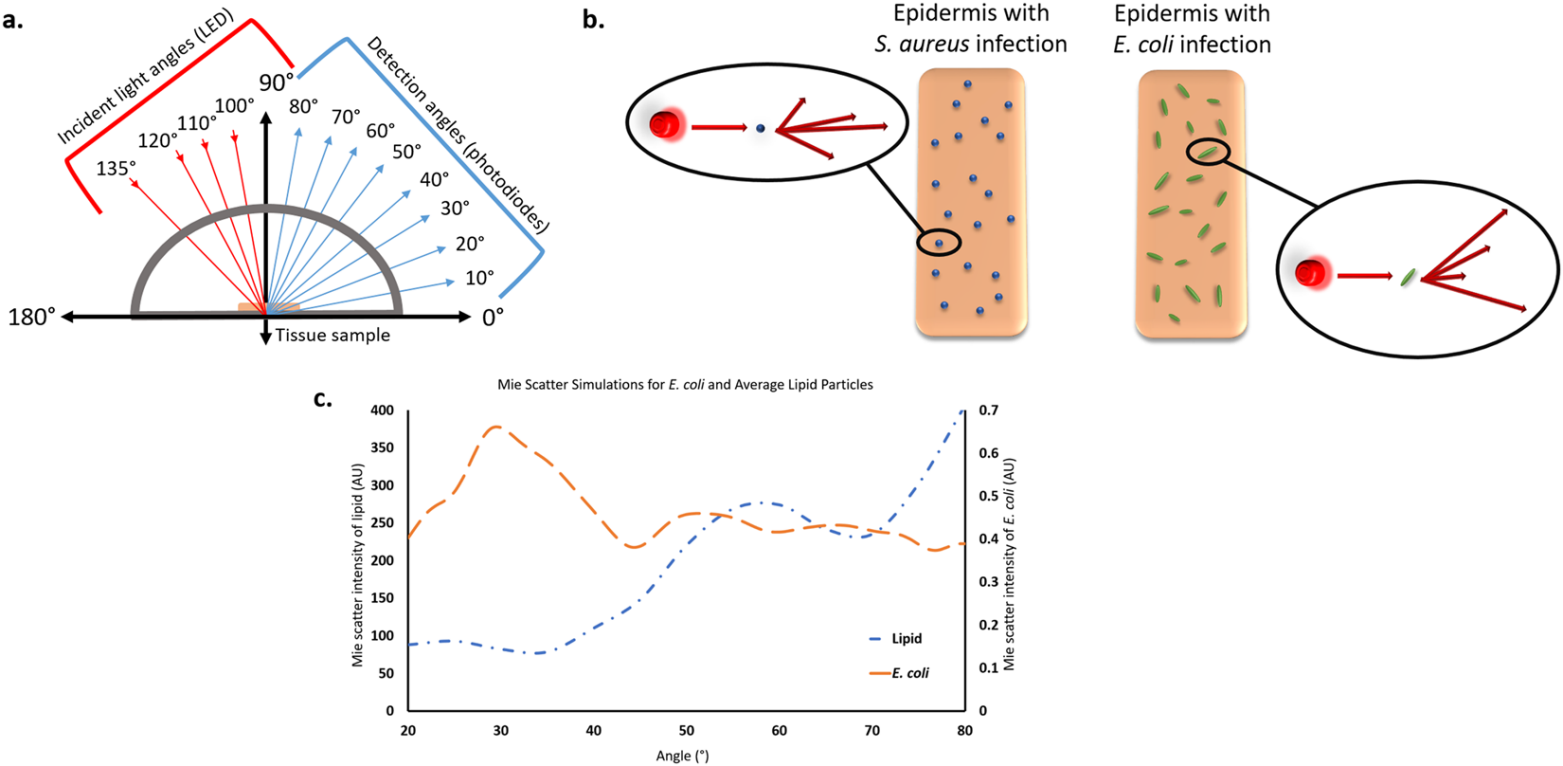
Schematic of Mie scatter spectra-based identification of bacteria. (**a**) Definition of incident light and detection angles for this work. All angles are defined relative to the tissue sample being placed on the plane from 0° to 180°. (**b**) A schematic representation of the concept of different bacterial species generating unique Mie scatter spectra. Based on the size (*r*), shape, and refractive index (*n*) of the bacterial species, Mie scatter intensities will vary across a range of detection angles, with unique spectra being produced by each species. In this example, *Staphylococcus aureus*, small cocci, results in one unique Mie spectrum, while *Escherichia coli*, medium bacilli, results in its own unique Mie spectrum across a variety of angles. (**c**) Mie scatter simulations conducted using the size and refractive index of *E. coli* versus those of typical lipid particles, much like those found in the skin. Simulations of expected Mie scatter trends show that *E. coli* should be distinguishable from lipid particles found within skin based on the use of a 650 nm light source resulting in a major peak at 30°.

Towards this goal, a custom stage was developed to monitor Mie backscatter from a tissue surface using a 650 nm LED at angles of 90°, 100°, 110°, 120°, and 135°, relative to the tissue surface. Mie scatter is detected at 10° increments from 10° to 80° to instantly obtain a full Mie scatter spectrum (angle definitions in Fig. 1a). Our novel device is capable of detecting infection on a tissue surface and the bacterial species responsible within seconds, without physically contacting the skin, and without using any reagents. Scale up of our device would allow a patient to simply scan skin to determine if an infection is present, and if so, the species of the infection.

Our goal was to develop a miniature prototype of such a device for rapid and mobile diagnosis of skin infection, as well as to determine the ability to differentiate species of bacterial infection.

## Results

### Experimental design

Mie scatter patterns change based on the refractive index (*n*), size (*r*), shape, and concentration of scattering objects; it was expected that differences in Mie scatter patterns could be used to identify specific bacterial species on skin surface (Fig. 1c). Mie scatter simulations were carried out to show the expected differences in scatter patterns for bacteria (ex. *E. coli*, *n* = 1.40, *r* = 2.5 µm) and lipids (*n* = 1.46, *r* = 10 µm) that would be found in the skin (Fig. 1c). Mie scatter simulations in Fig. 1b show that at 30° (120° if we have to add 90° to indicate back scattering) a major peak is expected for *E. coli*, but not for larger lipids that would be found in tissue.

**Figure 2:**
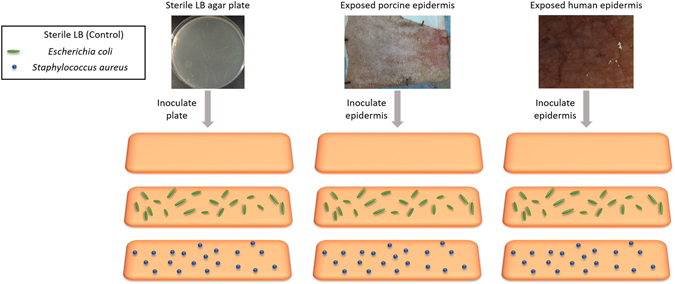
Experimental setup. Experimental design was based off of detecting two species of pathogenic bacteria from three surfaces. Lysogeny broth - Miller (LB-Miller) was used a control to directly compare to inoculations of bacteria in the same media. *E. coli*, *S. aureus*, and the sterile LB control were individually inoculated on each of three surfaces, LB agar plates, porcine epidermis, and human cadaveric epidermis. While the schematic representations shows that all samples had only the bacteria added via inoculation on them, porcine epidermis and human epidermis both have natural, commensal bacteria on their surfaces, but LB agar is sterile and should contain no additional bacteria on the surface.

Three different inoculations were used: *Escherichia coli, Staphylococcus aureus*, and a control of sterile lysogeny broth – Miller (LB-Miller), on three difference surfaces: LB agar, porcine epidermis, and human cadaveric epidermis (Fig. 2). Bacteria were shown to be grown successfully on skin surfaces and were identified after growth on the skin surface through selective and differential culture plating (Supplementary Figure S1).

### Device design

**Figure 3:**
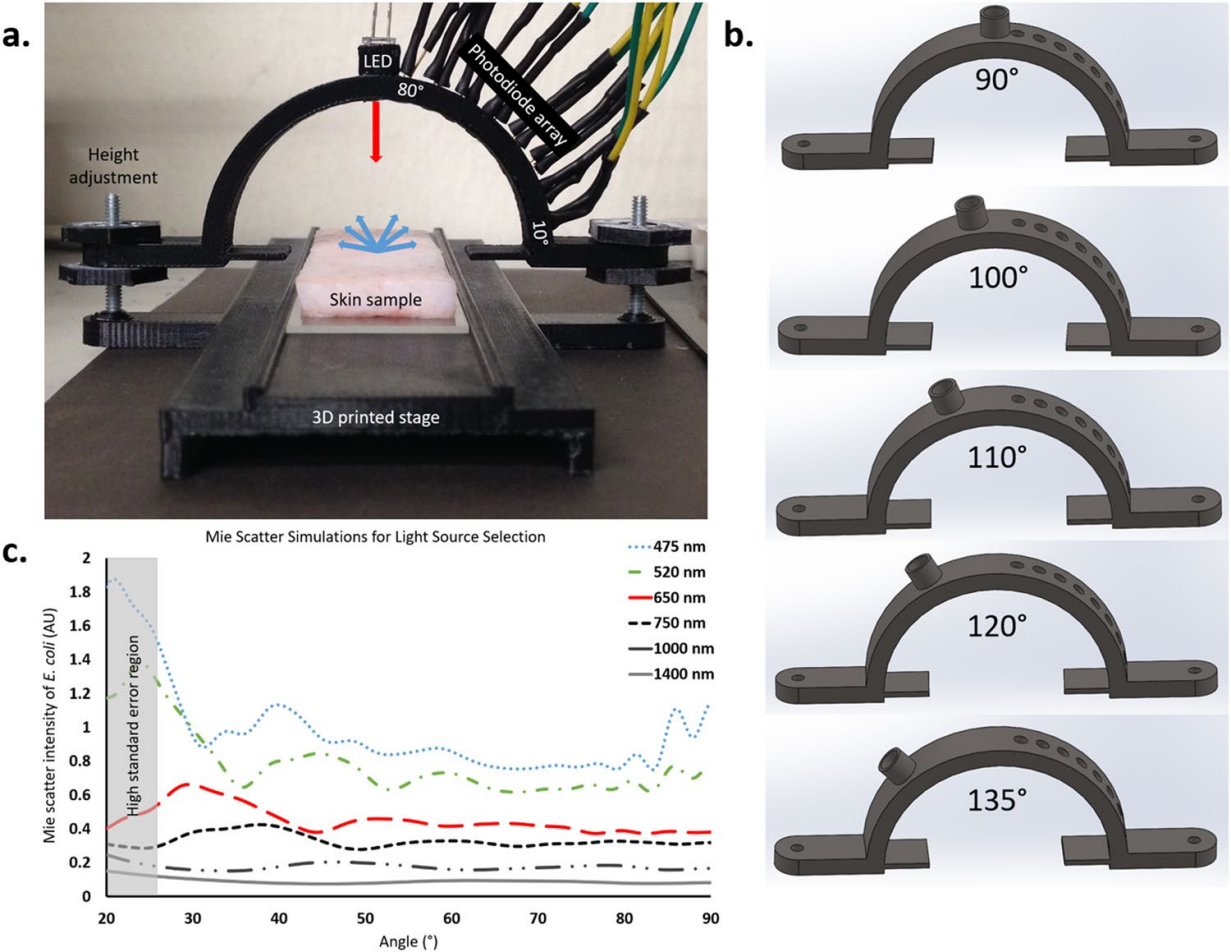
Device design. (**a**) The complete photodiode array used for data collection. The array is height adjustable to ensure analysis at the surface of each individual sample. A 650 nm LED illuminates the sample and Mie scatter spectra are detected by photodiodes at increments of 10° from 10° to 80° relative to the tissue surface. The incident angle of the LED is adjustable through individual attachments, due to space constraints on this miniaturized device. (**b**) Each of the five attachments used to change the incident light angle. All five attachments have identical sizes and photodiode locations, with the only difference being the angle at which the LED is held. Using these attachments, the incident light angle can be changed between 90°, 100°, 110°, 120°, and 135°. (**c**) Mie scatter simulations used to determine the optimal wavelength to use for the light source within this device. Simulations were conducted based off of parameters for *E. coli* and specifications of the device designed. Based on these simulations, a 650 nm light source was selected as the optimal incident wavelength.

Final device design is shown in Fig. 3a. A semi-circular angular array was 3D printed with insertion points for an LED and a series of PIN photodiodes (PDs). The array was attached to a 3D stage, designed to fit a standard microscope slide, in a manner that would allow adjustment of array height relative to the stage.

The angular array was designed to have insertion points for PDs at 10° increments from 10° to 80° and a single insertion point for an LED at either 90°, 100°, 110°, 120°, or 135°. Five arrays had to be designed to each hold the LED at a single angle due to space constraints, rather than having a single array to accommodate variable LED positions (Fig. 3b).

Following Mie scatter simulations (using MiePlot v4.2.11) a 650 nm red LED was used as a sole light source (Fig. 3c). Mie scatter simulations were performed for *E. coli* at wavelengths of 475 nm, 520 nm, 650 nm, 750 nm, 1000 nm, and 1400 nm (Fig. 3c). Wavelengths in the ultraviolet (UV) range were not considered due to their bactericidal capability and dangers of UV exposure to human skin. Side scatter results in large variability in data collected at 10° and 20°, due to the presence of hair follicles and imperfections in the topography of tissue samples. Light sources at 475 nm and 520 nm were not considered due to the major peaks of 21° and 24° (where noisy side scatter should be used), respectively. NIR wavelengths (750 nm, 1000 nm, and 1400 nm) were also ruled out due to low intensity major peaks and that the eventual goal of translation to smartphone would require an additional NIR light source. A 650 nm LED light source was selected based on a high intensity major peak at 30°, which avoids variability at low angles of detection, the designed device is able to detect this peak with a PD.

### SEM imaging of porcine skin infection model

**Figure 4:**
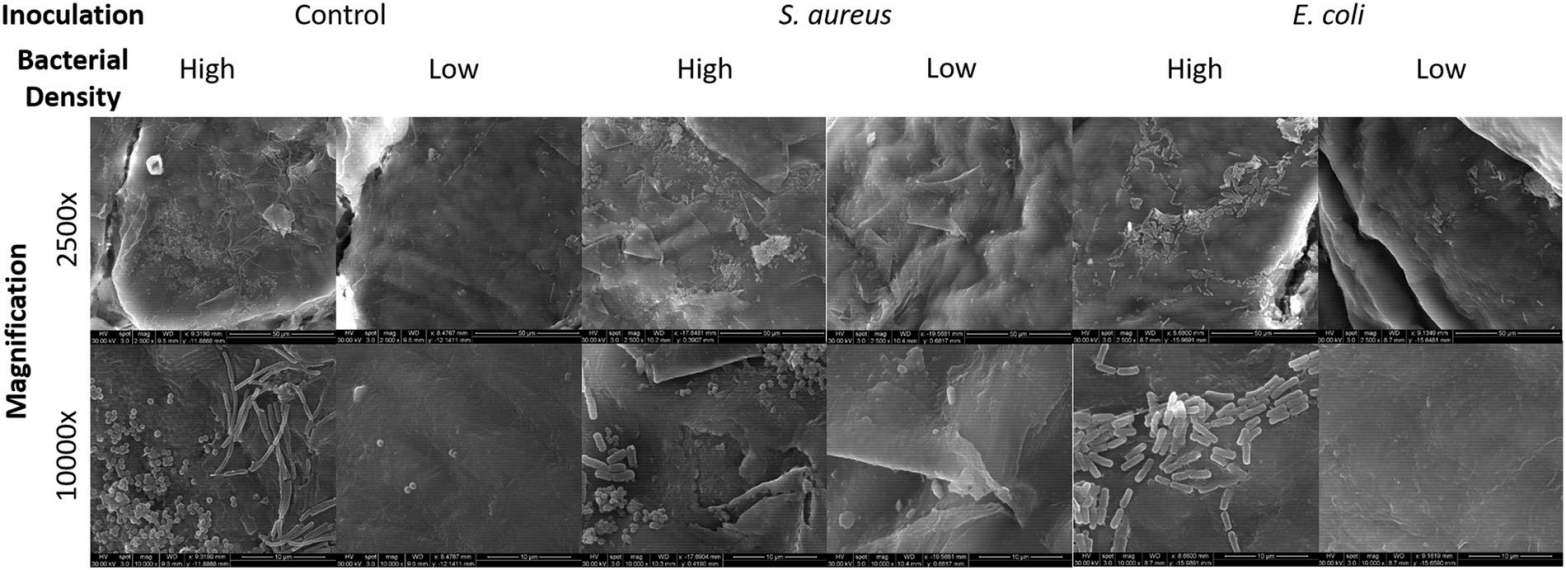
SEM images of porcine epidermis skin infection models. Images were captured at magnifications of 2,500 and 10,000 times by SEM. All images shown for each experimental group were captured from a single tissue sample, to show the diversity of the bacterial distribution, density, and concentration across each single tissue sample.

Figure 4 shows scanning electron microscope (SEM) images of the porcine skin infection model to show the distribution and density of bacteria as well as the presence of other scattering factors, such as dead cells, debris, and an uneven layering of skin cells. Control samples have a variety of bacterial species naturally on the skin surface and a diverse distribution and density of bacteria, much like the environment of natural skin. Samples inoculated with pathogenic bacteria (*S. aureus* or *E. coli*) have a diverse distribution and density of bacteria and it is obvious that commensal bacteria are still present on the surface of these infection models. Within each individual tissue sample, there are areas of high bacterial density, low bacterial density, large numbers of inoculated pathogens, a mixture of commensal and pathogenic bacteria, large numbers of commensal bacteria, and a variety of dead cells, debris, and microscopic topography of the layers of skin cells. Overall, SEM images show a well-mimicked, natural skin environment and skin infection environment.

### Identification of bacteria in pure culture

**Figure 5:**
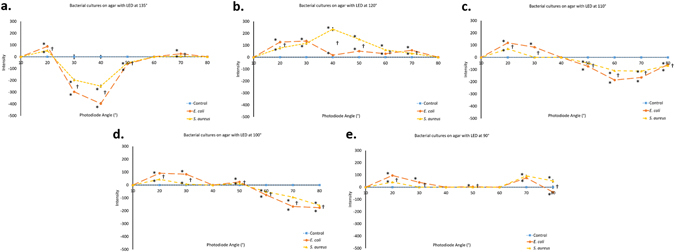
Identification of bacteria in a pure culture. (**a**–**e**) Data obtained from the photodiode array designed by scanning LB agar either sterile or with an inoculation of *E. coli* or *S. aureus*. Incident light angle was changed for each of the five data sets; 135° (**a**), 120° (**b**), 110° (**c**), 100° (**d**), and 90° (**e**). At each individual incident light angle, significant differences result between bacterial inoculation and control samples as well as between each species of inoculation; *E. coli* or *S. aureus*. As expected, the trend changes as the incident light angle changes, but distinct differences are maintained despite changing trends with changing incident angles. One data point is considered to be the average of 12 readings from each photodiode (3 s scan). Each of five agar samples was scanned at three unique locations for 3 s at each location. Error bars represent standard error of the mean. * is shown to the left side of each data point indicating significant difference (p < 0.05) between *E. coli* vs. control samples or between *S. aureus* vs. control samples. † is shown to the right side of each data point indicating significant difference (p < 0.05) between *E. coli* vs. *S. aureus* samples.

Bacteria grown on agar plates were tested using the designed photodiode array shown in Fig. 3a to determine if pure cultures of *E. coli* differed from those of *S. aureus*. Significant differences were detected between Mie scatter spectra across a variety of incident light angles of these two bacterial species grown on LB-Miller agar despite no obvious visual differences between cultures on agar (Fig. 5). Mie scatter spectra at each incident angle differ as expected, but at each angle significant differences exist between *E. coli* and *S. aureus* at every incident light angle. Significant differences (p < 0.05) were found between *E. coli* vs. control samples (marked *), *S. aureus* vs. control samples (marked *), and *E. coli* vs. *S. aureus* samples (marked †) (Fig. 5).

### Identification of bacteria on porcine epidermis

**Figure 6:**
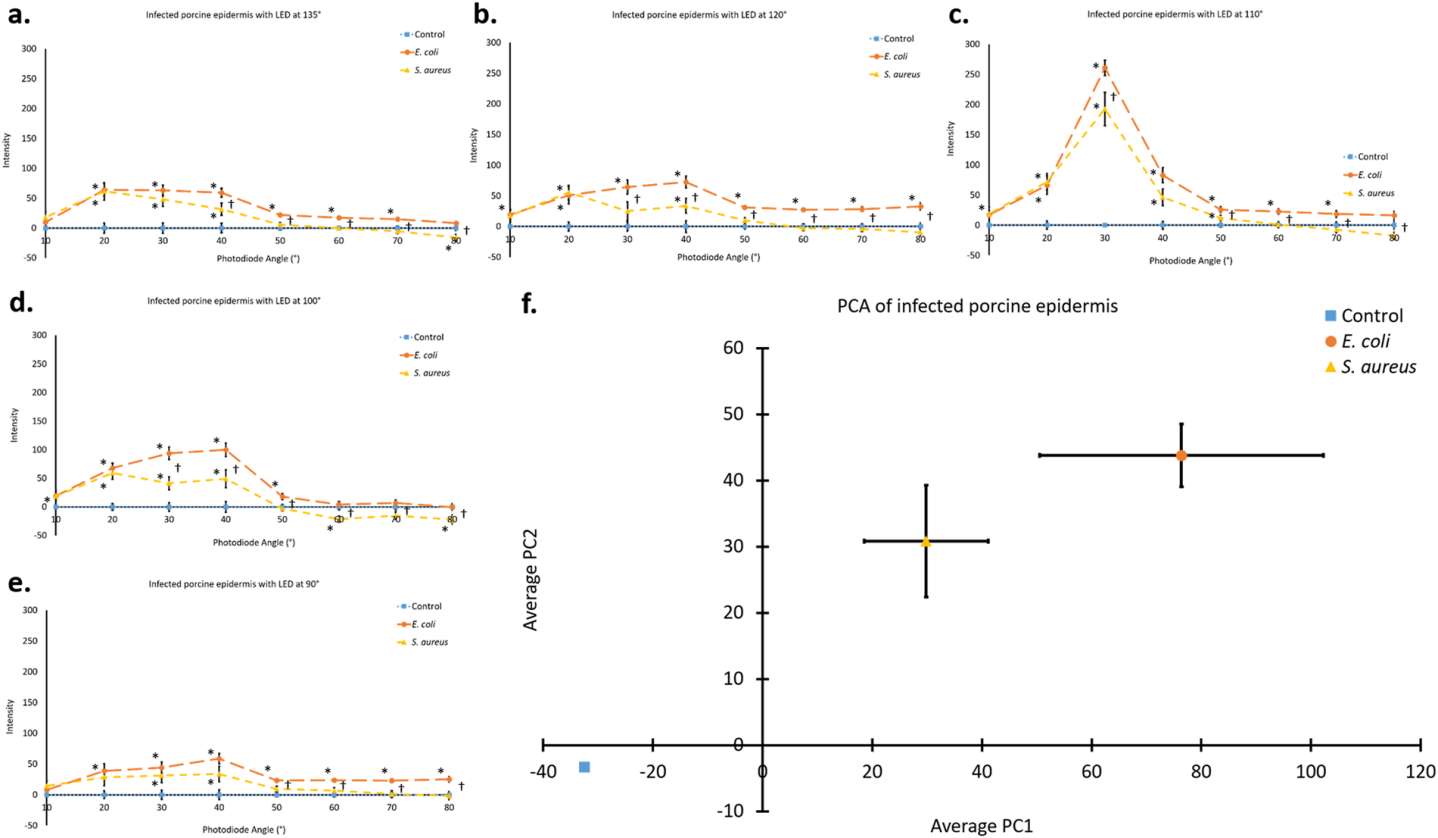
Identification of bacteria on a porcine epidermis model. (**a**–**e**) Data obtained from the photodiode array designed by scanning porcine epidermis samples inoculated with sterile LB, *E. coli*, or *S. aureus*. Incident light angle was changed for each of the five data sets; 135° (**a**), 120° (**b**), 110° (**c**), 100° (**d**), and 90° (**e**). At each individual incident light angle, significant differences result between bacterial inoculation and control samples as well as between each species of inoculation; *E. coli* or *S. aureus*. As expected, the trend changes as the incident light angle changes, but distinct differences are maintained despite changing trends with changing incident angles. One data point is considered to be the average of 12 readings from each photodiode (3 s scan). (**f**) Principle component analysis (PCA) of the data shown in (**a**–**e**) shows distinct differences between infections of the porcine epidermis with *E. coli* vs. *S. aureus* vs. only the commensal bacteria present on control samples. The two principle components shown, PC1 and PC2, account for 92% of the data shown in (**a**–**e**), 79% and 13% for PC1 and PC2, respectively. Each of five porcine epidermis samples was scanned at three unique locations for 3 s at each location. Error bars represent standard error of the mean, the control group in (**f**) has standard error of zero. * is shown to the left side of each data point indicating significant difference (p < 0.05) between *E. coli* vs. control samples or between *S. aureus* vs. control samples. † is shown to the right side of each data point indicating significant difference (p < 0.05) between *E. coli* vs. *S. aureus* samples.

Porcine epidermis samples inoculated with *E. coli* showed significantly different Mie scatter spectra than those inoculated with *S. aureus* (p < 0.05, marked †), despite the presence of natural, commensal bacteria on all porcine epidermis samples (Fig. 6). Mie scatter spectra between these species consistently differed across all incident light angles using the developed device (Fig. 6a–e). Significant differences (p < 0.05) were found between *E. coli* vs. control samples (marked *), *S. aureus* vs. control samples (marked *), and *E. coli* vs. *S. aureus* samples (marked †) (Fig. 6).

To summarize differences between Mie scatter spectra produced by each species, principle component analysis (PCA) (Fig. 6f) was carried out on the data shown in Fig. 6a–e. There are 40 dimensions (8 detection angles and 5 incident angles) in raw Mie scatter spectra. Figure 6a–e shows the mean Mie scatter intensities of 15 data points (five samples each at three different locations) for each given combination of incident and detection angle. PCA was conducted for each assay, not for the average values, and the mean principal component (PC) values with the standard errors were plotted on the PCA score plot. Using two principle components 92% of the data obtained was useful in explaining differences between the experimental groups (PC1 accounts for 79% of data, PC2 accounts for 13% of the data). Data obtained from an incident light angle of 110° was excluded from PCA due to the outlier effect the significantly higher magnitude of intensity at this angle (Fig. 6c). Significant differences (p < 0.05) exist between the presence of additional bacteria (an infection) and natural, commensal bacteria, as well as the two species of bacterial infections tested. Through PCA, significant differences were shown (p < 0.05) between PC1 for *E. coli* vs. that for control samples as well as for *S. aureus* vs. that for control samples.

### Identification of bacteria on human cadaveric epidermis

**Figure 7:**
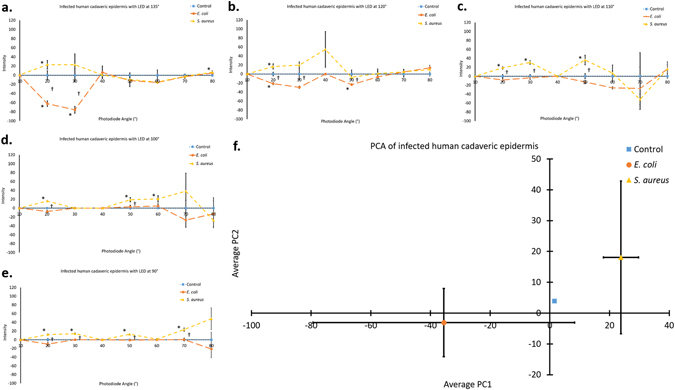
Identification of bacteria on a human cadaveric epidermis. (**a**–**e**) Data obtained from the photodiode array designed by scanning human cadaveric epidermis samples inoculated with sterile LB, *E. coli*, or *S. aureus*. Incident light angle was changed for each of the five data sets; 135° (**a**), 120° (**b**), 110° (**c**), 100° (**d**), and 90° (**e**). At each individual incident light angle, significant differences result between bacterial inoculation and control samples as well as between each species of inoculation; *E. coli* or *S. aureus*. As expected, the trend changes as the incident light angle changes, but distinct differences are maintained despite changing trends with changing incident angles. One data point is considered to be the average of 12 readings from each photodiode (3 s scan). (**f**) Principle component analysis (PCA) of the data shown in (**a**–**e**) shows distinct differences between infections of the human cadaveric epidermis with *E. coli* vs. *S. aureus* vs. only the commensal bacteria present on control samples. The two principle components shown, PC1 and PC2, account for 70% of the data shown in (**a**–**e**), 49% and 21% for PC1 and PC2, respectively. Adding an additional PC, PC3 would account for an additional 14% of the data shown in (**a**–**e**). Each of 2 human cadaveric epidermis samples was scanned at three unique locations for 3 s at each location. Error bars represent standard error of the mean, the control group of (**f**) has standard error of zero. * is shown to the left side of each data point indicating significant difference (p < 0.05) between *E. coli* vs. control samples or between *S. aureus* vs. control samples. † is shown to the right side of each data point indicating significant difference (p < 0.05) between *E. coli* vs. *S. aureus* samples.

Human cadaveric epidermis inoculated with *E. coli* showed significantly different Mie scatter spectra than those inoculated with *S. aureus* (p < 0.05, marked †), again, despite the presence of commensal bacteria on the skin. Mie scatter spectra differed between each inoculated species of bacteria and between the presence of infection and presence of only commensal bacteria, shown through control group of sterile LB inoculation (p < 0.05, marked *) (Fig. 7a–e). Error bars show standard error of the mean.

Again, to summarize differences between Mie scatter patterns produced by each bacterial species, PCA was carried out (Fig. 7f). In this instance, PC1 and PC2 accounted for 70% of data (49% and 21%, respectively). The introduction of a third dimension here, PC3, would account for a further 14% of the data. Significant differences (p < 0.05) are shown between both infected and control samples and each species of bacteria infecting the human cadaveric skin surface, *E. coli* and *S. aureus*, although the standard error is larger than those from porcine skin data.

### Lack of interference by common skin contaminants

An inoculation of body lotion was used to determine the ability of the designed device to detect only bacteria presence, rather than the presence of non-bacterial skin contaminants. Results are shown in Supplementary Figure S2. Only 10 out of 40 data points for the Mie scatter spectrum collected were significantly different (p < 0.05), but PCA showed that no significant differences exist overall between lotion coated samples and control samples.

### Determination of bacterial species on skin surfaces

Pure bacterial cultures were cultured on selective and differential media, specifically eosin methylene blue (EMB) agar, mannitol salt agar, and blood agar (TSA + 5% sheep’s blood). Results are shown in Figure S1a. As expected, *E. coli* did not grow on mannitol salt agar, and grew black with a green metallic sheen on EMB agar, showing that it is in fact *E. coli* growing. Also as expected, *S. aureus* did not grow on EMB agar, grew with beta hemolysis on blood agar, and turned the mannitol salt agar yellow, showing that it is in fact *S. aureus* growing.

Following inoculation of bacteria on porcine epidermis samples, the surface was stamped onto selective and differential media. Results are shown in Figure S1b. Samples inoculated with *E. coli* grew identically to pure cultures of *E. coli*, confirming that the inoculated *E. coli* did in fact grow on the surface of the porcine epidermis. Samples inoculated with *S. aureus* grew identically to pure cultures of *S. aureus*, confirming that the inoculated *S. aureus* did in fact grow on the surface of the porcine epidermis. Samples inoculated with sterile LB (control), showed signs of neither *E. coli* nor *S. aureus* growth to an extent comparable to samples inoculated with these bacteria. For all samples, commensal bacterial present on the skin prior to inoculation also grew, as expected, showing signs of low amounts of *E. coli* and *S. aureus*, which would be expected on skin samples.

### Swab culture for bacterial concentration

Swab culture concentrations of all experimental groups (control, *S. aureus*, and *E. coli*) were within the same order of magnitude. Control samples (after incubation) had an average bacterial concentration of 3.8 × 10^7^ CFU/cm^2^, *S. aureus* skin infection models had an average bacterial concentration of 2.2 × 10^7^ CFU/cm^2^, and *E. coli* skin infection models had an average bacterial concentration of 1.7 × 10^7^ CFU/cm^2^.

Swab culture concentrations of porcine skin samples prior to any inoculation were determined to be 2.3 × 10^5^ CFU/cm^2^.

### Summary of results

In summary, the device designed (Fig. 3) has been shown to detect differences in Mie scatter patterns between *E. coli*, *S. aureus*, and a control of sterile LB inoculation on agar (Fig. 5), porcine epidermis (Fig. 6), and human cadaveric epidermis (Fig. 7), while detecting no differences between control groups and common skin contaminants like body lotion (Figure S2).

## Discussion

Cultures of individual bacterial species were inoculated on porcine and human cadaveric epidermis samples to mimic infection. A unique trend can be detected between each bacterial species used in this study, showing that rapid identification of bacterial infection based on Mie scatter is possible for skin infection. The refractive index, size, and shape of individual scattering objects (here, bacterium) determine Mie scatter spectra, so it is expected that bacteria of different refractive indices, size, and shape (i.e. different species) result in different Mie scatter spectra. Notable differences between gram-negative bacilli bacterial species (*E. coli*) and gram-positive cocci bacterial species (*S. aureus*) is critical in a robust bacterial infection detection system. *S. aureus, Pseudomonas aeruginosa, Streptococcus pyogenes*, and *E. coli* are some of the most common pathogens in skin infection, and these bacteria differ in size, shape, and Gram stain, and subsequently refractive index.

It is clear from the SEM images shown in Fig. 4 that the bacterial distribution, density, and concentration are diverse across all experimental groups and across any given tissue sample, which mimics a real-life skin and skin infection environment. It is also clear from these SEM images that commensal bacteria are present on both control samples and infection model samples, and that there are no apparent differences between the distribution and density of bacteria between control samples and infection model samples. All images show that most areas of the skin samples have other scattering factors, such as dead cells and debris, mimicking a real-life skin and skin infection environment.

While differences were detected through simply scanning the surface of the skin and normalizing to healthy (not infected) skin, these differences become more distinct and allow for species identification when processed using PCA. Data collected from an incident light angle of 110° was excluded from PCA due to the high intensity of scatter at this angle relative to the intensity at all other incident light angles (Fig. 6c). It is believed that 110° incident light angle is the optimal angle for scatter from the topography of the skin (i.e. hair and other imperfections). The standard error of PC1 and PC2 are believed to be larger for human skin samples than those for porcine skin samples due to a combination of the small sample size (due to limited availability of human skin) and the storage of human skin for years in a frozen state and the thawing of the frozen tissue. Although freezing the skin helps to prevent decomposition of the skin, denaturation of the epidermis does occur between freezing and thawing of the tissue. Using this technique, the device developed was able to determine not only the presence of an infection, but further the species of bacteria responsible for the infection. Immediate and specific diagnosis of the species of infection would help to reduce the need for the use of broad spectrum antibiotics, which contribute greatly to antibiotic resistance, instead allowing for the use of narrow spectrum antibiotics. While this device is likely unable to determine resistant versus susceptible strains within a species, the use of this device does allow for the potential to bypass the initial steps of identifying the bacteria responsible for the infection, instead allowing for specific culture and genetic tests on resistance to be carried out immediately.

Bacterial concentrations for all experimental groups were within the same order of magnitude with no substantial differences between concentrations on control samples and infected samples. Despite no differences in bacterial concentration between control and infected samples, our device was successful in determining the presence and species of an infection when a pathogen was added to the surface and the commensal bacteria already present.

The lack of change in concentration despite the addition of pathogenic bacteria shows that, much like a realistic skin infection, the pathogenic bacteria overcame the commensal bacteria on the surface of the skin through competitive inhibition. Competitive inhibition occurs in a skin infection when pathogenic bacteria are introduced and overgrows to overcome the commensal bacteria on the skin surface, so the bacterial environment on our skin infection model appears to be realistic.

Despite the fact that bacterial concentrations on the skin naturally vary greatly, the World Health Organization has summarized that typical concentrations of commensal bacteria range from 1 × 10^4^ CFU/cm^2^ to 4.6 × 10^6^ CFU/cm^2^, with location on the body being a major contributing factor to this variation^28^. Prior to inoculation and incubation, the porcine skin model used showed comparable numbers of bacteria (2.3 × 10^5^ CFU/cm^2^).

When pathogenic bacteria are introduced to skin and dominate over commensal bacteria (thus triggering symptoms), the total bacterial concentrations (pathogenic plus commensal bacteria) are likely to be higher than the above values and vary based on the location on the skin and degree of symptoms. The swab culture concentrations of *S. aureus* and *E. coli* infection models, 2.2 × 10^7^ CFU/cm^2^ and 1.7 × 10^7^ CFU/cm^2^, respectively, are within a reasonable range of total bacterial concentrations estimates for general skin infection models. The exact limit of detection could be better determined using models of skin infection that are specific to regions of the skin, since bacterial concentration is known to vary based on the exact region of skin in question, which is potential future work in the development of this device.

The fact that our device was shown to detect the presence and species of a pathogen despite a lack of difference in concentration of bacteria between control and infected samples shows that our device is promising for monitoring skin infection. Our device was able to detect a difference in bacterial species dominating a tissue sample rather than a difference in bacteria number on the surface.

Through the use of a porcine skin model and human cadaveric skin, we have shown that our device is capable of detection of pathogenic bacteria on skin, and therefore should have promising results in a clinical setting. The data in this study was acquired in as little as three seconds, so an immediate analysis could be performed clinically.

With the technology developed through this work, patients can monitor infection at home and be alerted to seek medical attention as soon as an infection begins. The developed technology could be used in areas where immediate access to a physician is not possible, so infection can be diagnosed before it is able to become tissue or life threatening infection.

Variation seen in the presented data is likely due to the presence of commensal bacteria on skin. Skin has high numbers of non-pathogenic bacteria; this system does pick up signals from these commensal bacteria. Variation due to commensal bacteria is reduced in this study by normalizing data to control samples. In a clinical setting, data could be normalized to an area of healthy skin to reduce variation due to natural, healthy bacteria presence.

## Conclusion

A portable and inexpensive device was successfully designed to diagnose pathogenic bacteria presence on agar, a porcine skin model, and on human skin. Species-specific detection of bacterial infections on tissue samples is possible with the angular photodiode array developed in this study. Bacterial species that have different shapes, sizes, and Gram stains (and thus refractive indices) show unique trends across various angles of detection. Information obtained from our device can determine if an infection is present, and, if so, the responsible bacterial species and therefore the ideal initial antibiotic treatment. Common skin contaminants (e.g. body lotion) have little to no impact on the Mie scatter spectra from tissue samples, which is promising for translation to clinical use.

## Methods

### Study Design

Study groups in this controlled laboratory experiment include *Escherichia coli*, *Staphylococcus aureus*, and a control of sterile LB-Miller medium on three surfaces; LB-Miller agar, porcine epidermis, and human cadaveric epidermis (Fig. 2). Mie scatter spectra were collected using a custom angular photodiode array at 10° increments from 10° to 80° (Fig. 3a). Each sample was analyzed at three separate locations for a representative collection of scatter from an entire surface. Each of the three locations was analyzed with the LED light source at five different angles relative to the surface; 90°, 100°, 110°, 120°, and 135° (Fig. 3b). Samples were randomly assigned an inoculum and researchers were not blinded.

### Bacteria solutions

*Escherichia coli* K12 (Sigma-Aldrich, St. Louis, MO, USA) and *Staphylococcus aureus* (ZeptoMetrix, Buffalo, NY, USA) were individually cultured in lysogeny broth (LB) Miller’s formula (Molecular Biologicals International Inc, Irvine, CA, USA) at 37 °C for 8 hours. Bacteria were freshly cultured prior to each experiment. All bacterial cultures were grown to maximum concentration (10^8^ CFU/mL). Concentration was determined through serial dilution and plate counting. Sterilized LB was used as a control to directly compare to bacteria grown in the same broth.

### Bacteria cultures on agar

Freshly grown cultures were plated on LB-Miller agar plates (BIO5 Media Facility, Tucson, AZ, USA) by uniformly spreading 400 µL of a bacteria culture across the surface as a puddle to avoid texture due to introducing an inoculating loop or needle. Cultured agar was cut to approximately 1.5 cm by 5 cm after 8 hours of incubation at 37 °C and placed on microscope slides with care taken to avoid disrupting the surface of the agar. Pure bacteria cultures on agar were analyzed using the photodiode array described.

### Porcine skin

Porcine skin (University of Arizona Food Products and Safety Laboratory, Tucson, AZ, USA) was acquired immediately following slaughter. Hair was shaved from the skin surface, exposing the epidermis. Samples were washed with water to remove excess dirt and cut into approximately 1.5 cm by 5 cm rectangles. Samples were then randomly divided into experimental groups and inoculated with 150 µL of either *E. coli*, *S. aureus*, or sterile LB-Miller medium, all of which were spread evenly across the surface of the skin with an inoculating needle. Samples were sealed with Parafilm M (Bemis Flexible Packaging, Oshkosh, WI, USA) in individual petri dishes and incubated for 8 hours to allow bacterial growth on the sample.

Rather than bacterial inoculation, alternative porcine dermis samples were coated with body lotion (St. Ives Skin Renewing Collagen Elastin, Unilever, Englewood Cliffs, NJ, USA), a common skin contaminant. Preparation and analysis of lotion coated skin samples was identical to that of bacteria inoculated samples, with the exception of inoculum.

### Human cadaveric skin

Human cadaveric skin was acquired following a period of deep freeze from the ankle region of one male individual. Cadaveric skin samples were prepared into identical samples as porcine skin. The epidermis remained intact for all samples, but excess hair was shaved from the surface where necessary. Samples were allowed to rest at room temperature following inoculation of 150 µL of *E. coli*, *S. aureus*, or LB (Miller) broth before analysis. Human skin samples were not incubated as porcine skin samples were due to the melting temperature of the lipids in the human skin, instead, bacteria were allowed to adjust to the surface during a 15-minute resting period. All experiments with human cadaveric skin samples were performed in accordance with relevant guidelines and regulations. Since these samples were cadaveric tissues, approval by the institutional review board of the University of Arizona was waived. All samples were from the willed body program at the University of Arizona College of Medicine.

### SEM imaging

Porcine skin samples were imaged via SEM at 30 kV. Three samples from each experimental group were imaged, with three locations imaged on each sample, and images taken at magnifications of 2,500 and 10,000 times at each location, to get a representation view of the entire surface. Figure 4 shows images from a single sample for each experimental group, to show the diversity of the surface of a single skin sample. Samples were fixed in paraformaldehyde and dehydrated with ethanol and 1,1,1,3,3,3-hexamethyldisilazane (HMDS) (Acros Organics, New Jersey, USA). Samples were gold sputter coated and imaged by FEI Inspec S system.

### Selective and differential culture plating

To show that the inoculated bacteria did in fact survive and grow on the tissue surfaces, following inoculation and growth of bacteria, skin surfaces were stamped on selective and differential agar plates to identify bacteria. Eosin methylene blue (EMB) agar (Hardy Diagnostics, Santa Maria, CA and Springboro, OH, USA) was used to identify *E. coli*, based on the growth of *E. coli* as black with a green metallic sheen on this agar. Both blood agar (BIO5 Media Facility, Tucson, AZ, USA) and mannitol salts agar (Hardy Diagnostics, Santa Maria, CA and Springboro, OH, USA) were used to identify *S. aureus*, based on the growth of this bacterium with a distinguishable hemolysis on blood agar and the survival of this bacterium on mannitol salts agar, combined with the color change of the agar to yellow.

### Swab culture for bacterial concentration

Using sterile cotton swabs, each of three tissue samples in each experimental group were swabbed for bacteria. The surface of each tissue sample was thoroughly swabbed by rotating the sterile cotton swab on the surface for about one minute. The swab was then placed in 1 mL of of sterile DI water and vortexed twice for 30 seconds each time. Serial dilutions of this stock solution were spread plated on LB agar and concentration was determined via bacterial culture plate counts. The area of each sample was measured to result in a concentration in terms of CFU/cm^2^. Following this same technique, bacterial concentrations for skin samples prior to inoculation and incubation (after hair removal and rinsing) were determined as well.

### Mie scatter detector

Directly following incubation of tissues, samples were placed on a standard microscope slide with care taken to not disrupt the surface of the skin. A 3D printed stage held the microscope slide in a fitted channel to be analyzed by a custom photodiode array. A 650 nm red LED light source illuminated the tissue with an array of PIN photodiodes detecting scatter off of the tissue sample at 10° increments from 10° to 80° relative to the tissue surface (Fig. 3a). The incident light angle was changed with each tissue sample being analyzed at each of five incident angles; 90°, 100°, 110°, 120°, and 135° (Fig. 3b). All photodiodes were used in photovoltaic mode (optimum mode for PIN PDs) and connected to LM324 quad op-amps to amplify current outputs. Amplified signals were sent to the analog inputs of an Arduino Mega 2560 microcontroller, providing an angular spectrum. Array height was adjusted prior to each measurement, so the array was level with the surface of each tissue sample.

### Data collection

Each tissue sample was analyzed at three different locations. Photodiode (PD) readings were collected every 250 ms and averaged over three seconds (12 data points per PD angle at each LED angle at each of three location on a sample; 1,440 data points per tissue sample). For each sample location and LED angle, photodiode readings were averaged at each angle, and standard error of the average of each 3 s reading was calculated over three different tissue locations of multiple samples for the purpose of error bars.

### Mie scatter simulation

Mie scatter simulations were carried out using MiePlot v4.2.11. For lipids, the following parameters were used^25^: particle radius (*r*) = 10 µm, refractive index (*n*) = 1.46, refractive index of medium (water) = 1.33, wavelength = 650 nm, and size distribution was assumed to follow a normal distribution with 10% standard deviation. For individual *E. coli*, the following parameters were used^23, 25^: particle radius (*r*) = 2.5 µm (hydrodynamic dimensions of 5 µm × 1 µm), refractive index (*n*) = 1.40, refractive index of medium (water) = 1.33, wavelength = 650 nm, and size distribution was assumed to follow a normal distribution with 10% standard deviation. To determine the appropriate light source for the device, simulations were conducted based on *E. coli*, varying only wavelength of the light source from 475 nm to 1400 nm.

### Data analysis

Data was initially analyzed using Microsoft Excel. Data was normalized to the control group (LB inoculation) by subtracting the average over 3 s of PD readings at each angle of the control from that of the experimental group. The goal in doing this was to eliminate noise due to commensal bacteria present on the skin prior to bacterial inoculation (infection). Data was then plotted within the program to display differences caused by the inoculation of bacteria on the sample. Student t-test assuming unequal variance was used to determine statistical significance with p < 0.05 being considered significantly different.

### Data analysis (PCA)

Due to the complex interactions between bacteria and the many cells types and macromolecules present in porcine and human skin, data collected from both skin types was further analyzed using principle component analysis (PCA). The Unscrambler v9.7 was used for PCA of the average of 12 PD readings averaged over three locations on each sample at each angle for each of five LED angles. Student t-test assuming unequal variance was used to determine statistical significance with p < 0.05 being considered significantly different.

### Data availability

All data generated or analyzed during this study are included in this published article (and its Supplementary Information files).

## Electronic supplementary material

Supplementary Info
