# Supplementary material for: Mie scatter spectra-based device for instant, contact-free, and specific diagnosis of bacterial skin infection
Source: Sci Rep. 2017 Jul 6;7:4801. doi: 10.1038/s41598-017-05061-1 (PMC5500527; doi:10.1038/s41598-017-05061-1)
Supplement: Supplementary file 1 — Supplementary Info [file 41598_2017_5061_MOESM1_ESM.pdf]

SUPPLEMENTARY INFORMATION

Title: Mie scatter spectra-based device for instant, contact-free, and specific diagnosis of bacterial skin infection

Authors: Robin E. Sweeney, Elizabeth Budiman, and Jeong-Yeol Yoon

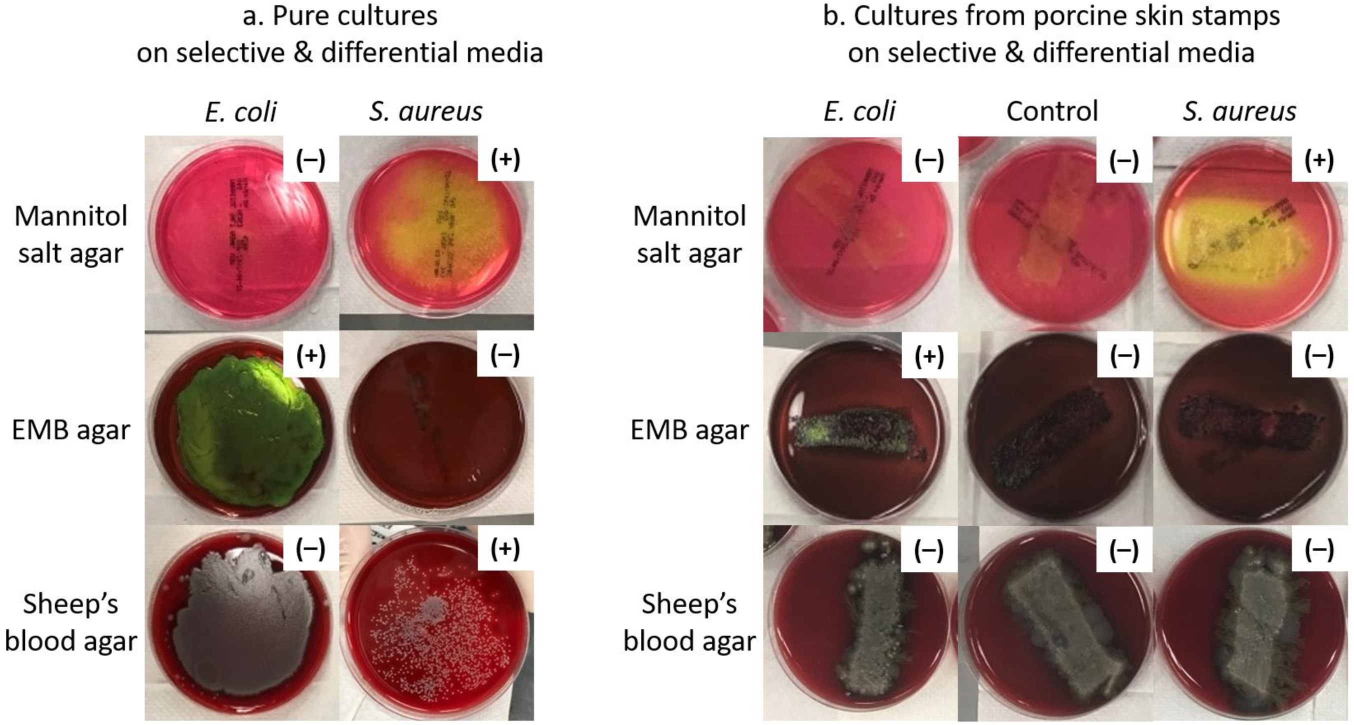

Supplementary Figure S1: Selective and differential culture of bacteria. a) Pure cultures of bacteria plated on mannitol salt agar, eosin methylene blue (EMB) agar, and sheep's blood agar, showing expected results for each bacterial species on each agar. Mannitol salt agar tests for *S. aureus* presence, as this bacterium grows on the agar (unlike most bacterial species) and turns the agar yellow. EMB agar tests for *E. coli* presence, as this bacterium grows on the agar (gram positive species do not) and grows as black colonies with a green, metallic sheen. Sheep's blood agar tests for *S. aureus* presence, as this bacterium grows with β-hemolysis. (+) shows the expected result for a selective and differential culture that contains the bacteria being tested for. (-) shows the expected result for a selective and differential culture that does not contain the bacteria being tested for. b) Bacterial cultures that result after stamping the epidermis of porcine skin samples on the agar after allowing the inoculated bacteria to grow for 8 hours on the porcine skin sample. Results confirm that the bacteria inoculated onto the surface of the porcine epidermis did in fact grow and thrive as the predominant species of bacteria on the skin surface, despite the presence of commensal bacteria. Due to the abundance of growth on the sheep's blood agar, hemolysis cannot be seen in any culture.

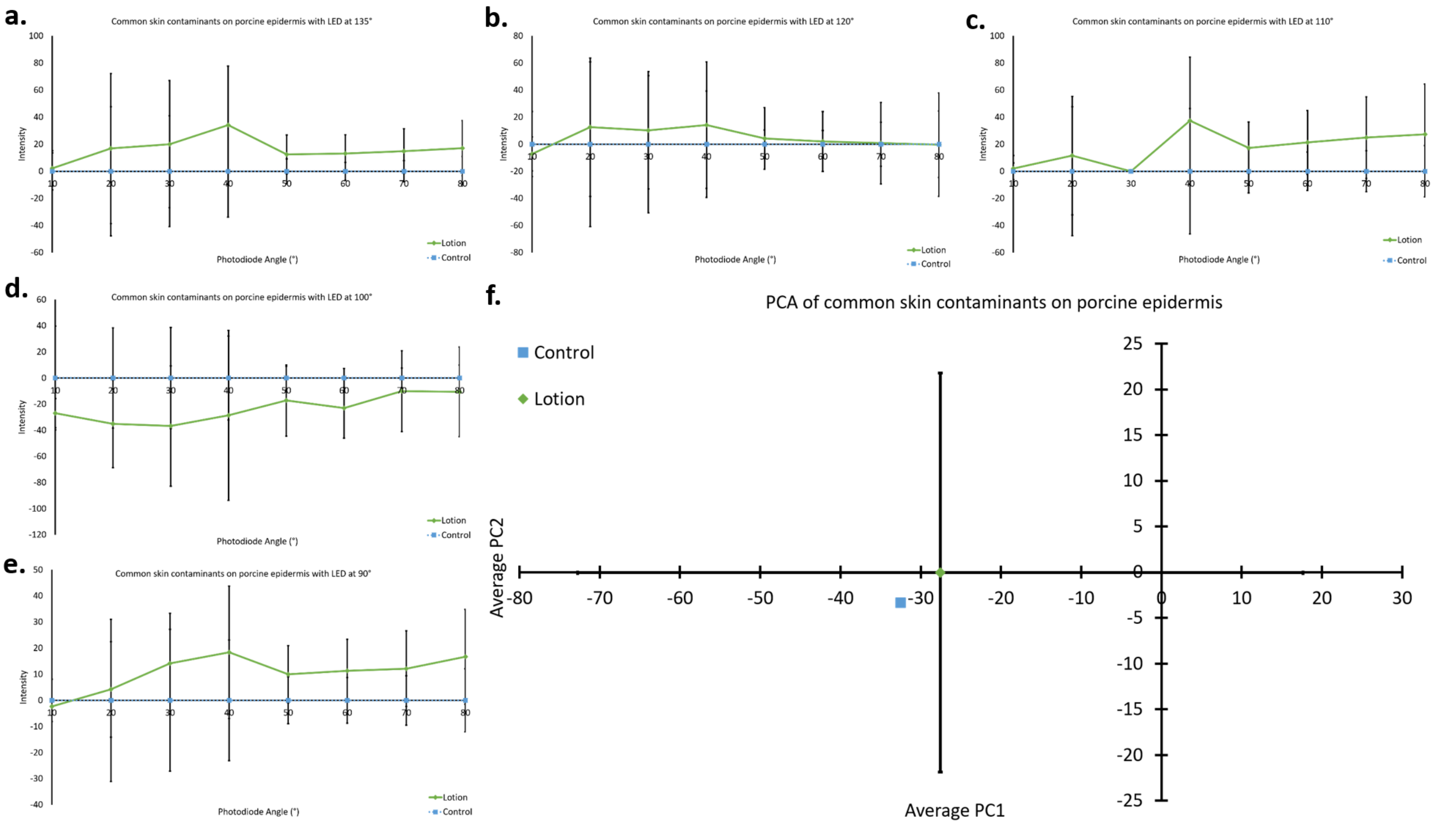

Supplementary Figure S2: Common skin contaminants on porcine epidermis. a-e) Mie scatter spectrum obtained from lotion-coated porcine epidermis samples. Only 10 out of 40 data points were significantly different ($p < 0.05$) from control samples (no inoculation). f) PCA showed that overall, no significant differences were detected between lotion-coated porcine epidermis samples (common skin contaminant) and control samples. The lack of interference of common skin contaminants with the diagnostic abilities of this device are promising for translation to clinical use, where patients may or may not have lipid-based skin contaminants (lotions, sunscreens, etc.) on the surface of their skin.
